# Event and Comment

**Published:** 1921-07

**Authors:** 


### DENTAL REGISTER

THE MAGAZINE OF DENTISTRY

Vol. LXXV JULY, 1921 No. 7

### EVENT AND COMMENT

**Are Dental Diseases Curable?** One of the more important problems that dentists have to consider is the question of how best to treat dental diseases which are liable to become systemically involved. Local injuries are liable to systemic invasion with serious consequences. Consequently, all localized diseases, however insignificant they may seem, should be adequately treated in their primary or initial stages, if it is practicable to administer the remedies. But we very well know the ordinary or routine treatments are not given the prompt and safe treatments needed to insure healthy tissues, and this prevents tedious and often serious and complicated wounds or radical surgical operations. This statement has only to be cited to recall the many bad results witnessed as a result of needless or serious diseases, or the bad wounds resulting from slight but neglected wounds with consequential results. The important matter, therefore, which we must guard carefully is whether the ordinary dental injuries are so inconsequential that they are not liable to become important factors in disease, and so become cases of greater consequence than we often realize. When we shall know the consequences of the usual and so-called trivial injuries, we shall probably safeguard our bodies more diligently than now, because we can foresee evil consequences from trivial injuries.

We can scarcely approach this phase of this subject without recalling the innumerable chances for injury and dangers to which the teeth and gums are threatened by the customary wear and tear incident to the mastication of the food. The teeth are injured, sometimes irreparably, in functional use; and the other tissues are seriously injured and may be infected beyond repair by careless and thoughtless medication or slight wounds. With the best care we can give the teeth and gums injuries and infections are necessarily possible as well as probable. Is there anything we can or should do, that we have not earnestly tried to prevent the continued injuries and losses to these important organs and their incidence to oral diseases?

When we discovered the manner in which the teeth were lost by carries, and when we found why the alveolar process and gums dissolved away by slow infection, we thought by medication and surgical intervention we should be able to check these diseases, or possibly to eradicate them entirely. To our sorrow we have only partially intercepted these influences, and it seems that new dangers continue to threaten our peace of mind with a new crop of worries, and we are beginning to believe that until we can stop infection in the mouth entirely, we shall never be able to control diseases anywhere in the body. Now we are told by our medical confrers that we must stop all possible infection in the mouth, so that there may be no serious infection in any of the vital organs of the body. The hard calcified teeth and their contiguous tissues must be steril, even though it means edentulous jaws and impaired masticating functions. A serious calamity, but it may be necessary to prevent a more consequential catastrophy, even the loss of life. If infected teeth are such a serious menace, we must face this issue and do our part to prevent, if practicable, such a lamentable consequence.

Because of our long and devoted efforts to preserve intact the normal dental functions, we are loth to believe the conservative art of our profession is destined to fail in accomplishing our objective, and we are hoping our science and skill will yet show us the way out, and that the dental profession will yet establish a clean bill of health, and that comfort and good health shall result because mouth health is a practicable achievement in our science and art. May it not be possible that we have not yet exhausted our technical and scientific resources in our effort to stop decay of the teeth and the consequent requirements for inhibiting infections of the oral tissues? We haven't yet fully studied our problem of prevention of disease and its physiologic and pathologic bearings on this problem. If we are assured that dental infection is wrong and intolerable, it would seem logical to stress our endeavors to the limit to make it impracticable; because the teeth and adjacent environments must be absolutely healthy. This is, and has been, our ideals, and we have aimed to do this in endeavors. If we have not secured these results it may be because we have blundered in the methods we have adopted to reach the objective.

This means a big job, but it is sufficiently important to get our best men to work earnestly about its accomplishment. Do we know our anatomical and physiological facts well enough to start the work; and are we sure we realize the bacteriological and pathological bearings needed to enable us to understand the progressive steps in disease invasion? These and all the other factors which make up the whole destructive phenomina will help us to learn how to prevent disease; and also how to repair injuries, and to cure diseases so that they shall not constitute disease hazards but remain helpful and salutary though injuried.

Is it not practicable to repair diseased and injured dental members so that they will restore their functions and their health by more exact knowledge of the healing processes without excessively radical treatments? If this can be accomplished it would certainly be worth while doing. The effort seems plausible.

